# Light Fields for Face Analysis

**DOI:** 10.3390/s19122687

**Published:** 2019-06-14

**Authors:** Chiara Galdi, Valeria Chiesa, Christoph Busch, Paulo Lobato Correia, Jean-Luc Dugelay, Christine Guillemot

**Affiliations:** 1 Department of Digital Security, EURECOM, 06560 Sophia Antipolis, France; jean-luc.dugelay@eurecom.fr; 2 Hochschule Darmstadt, 64295 Darmstadt, Germany; christoph.busch@ntnu.no; 3 Department of Information Security and Communication Technology, Norwegian University of Science and Technology, 2815 Gjøvik, Norway; 4 Instituto de Telecomunicacoes/Instituto Superior Tecnico, Universidade de Lisboa, 1049-001 Lisboa, Portugal; plc@lx.it.pt; 5 Institut National de Recherche en Informatique et en Automatique, 35042 Rennes, France; christine.guillemot@inria.fr

**Keywords:** survey, light field, face analysis

## Abstract

The term “plenoptic” comes from the Latin words plenus (“full”) + optic. The plenoptic function is the 7-dimensional function representing the intensity of the light observed from every position and direction in 3-dimensional space. Thanks to the plenoptic function it is thus possible to define the direction of every ray in the light-field vector function. Imaging systems are rapidly evolving with the emergence of light-field-capturing devices. Consequently, existing image-processing techniques need to be revisited to match the richer information provided. This article explores the use of light fields for face analysis. This field of research is very recent but already includes several works reporting promising results. Such works deal with the main steps of face analysis and include but are not limited to: face recognition; face presentation attack detection; facial soft-biometrics classification; and facial landmark detection. This article aims to review the state of the art on light fields for face analysis, identifying future challenges and possible applications.

## 1. Introduction

This survey aims not only to collect and review studies showing how face analysis techniques can be adapted to the new light-field paradigm, but also to discuss the advantages of the use of light fields for face analysis compared to classical 2D imaging. The surveyed works are selected according to the following criteria: (i) the work deals with the use of light fields for face analysis; (ii) the work is published in peer-reviewed journal or conference proceedings.

Articles were retrieved thanks to Elsevier’s abstract and citation database, namely Scopus. The advanced search tool allows identification of articles based on keywords and performance of analysis on the retrieved data. The keywords used in this case included: “light field”; “Lytro”; “Raytrix”; “plenoptic”; “face recognition/detection/landmark/liveness/presentation attack detection”. No time range limits were imposed. The obtained list of papers was manually checked to exclude false matches (6 papers). Along with the articles retrieved from Scopus, all co-authors of this article contributed in adding any paper of interest not already included in the provided list.

**Figure 1:**
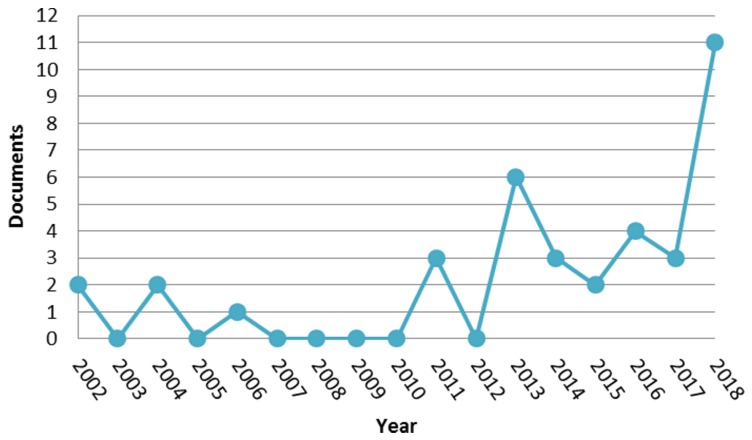
Graph of scientific papers published per year in the period 2002–2018 matching keywords for light fields and face analysis. From Scopus (https://www.scopus.com).

Figure 1 illustrates the number of published papers by year, in the period 2002–2018, about light fields for face analysis.

The graph shows that first attempts of introducing the concept of “light fields” for face analysis were made in 2002 [1,2] and 2004 [3,4]. The authors of these works used classical 2D RGB images to create a light-field-inspired object to address face pose variation. The first work using images captured by an actual plenoptic camera and thus using actual light-field face images was only published in 2013 [5].

The remainder of the survey is organized as follows: Section 2 introduces the light-field function and the novel features provided by the images captured by the devices implementing such technology, and the publicly available light-field face databases; Section 3, Section 4 and Section 5, review articles addressing the use of light fields for face landmark detection, face recognition, and face presentation attack detection, respectively. Section 6 discusses the main findings and implications for future research.

## 2. Background

### 2.1. Light Fields

**Figure 2:**
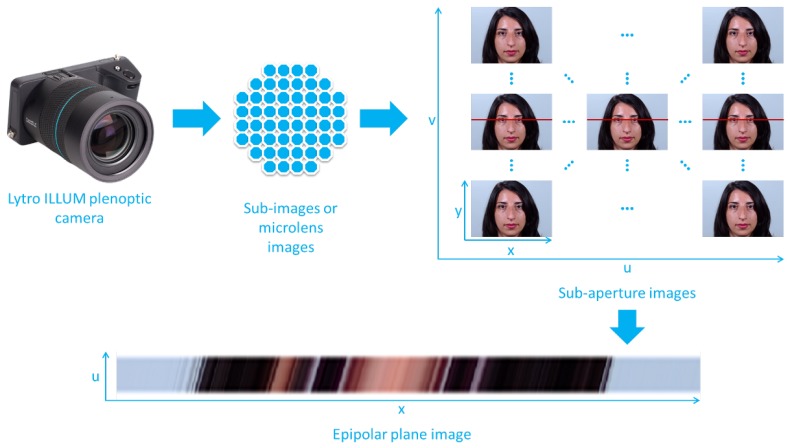
Epipolar plane image: process to obtain an epipolar plane image.

Light-field imaging has been recently gaining in popularity due to its interest for a variety of computer vision applications, such as 3D modelling, object detection, classification, or recognition, with applications in computational photography, augmented reality, light-field microscopy, medical imaging, 3D robotics, and also biometric recognition. Light fields represent the radiance of light rays emitted by any point in a 3D scene along different orientations that is generally described by a 7-parameters function: L(x,y,z,θ,ϕ,λ,t), where (x,y,z) are the 3D coordinates of the reference point, (θ,ϕ) are its angular coordinates, the point is observed at a particular wavelength λ and at a particular time *t*. Most of the plenoptic devices can collect only still images in visible spectrum, thus the parameters λ and *t* are fixed and often omitted. In some works ([6,7]), the authors adopt a different notation: the radiance is represented by a function L(x,y,u,v) of 4 parameters, where (u,v) denote the angular or view coordinates (corresponding to different orientations of the light rays), and where (x,y) denote the spatial or pixel coordinates. A light-field image can therefore be seen as capturing an array of viewpoints (called sub-aperture images, see Figure 2) of the scene with varying angular coordinates *u* and *v*.

**Figure 3:**
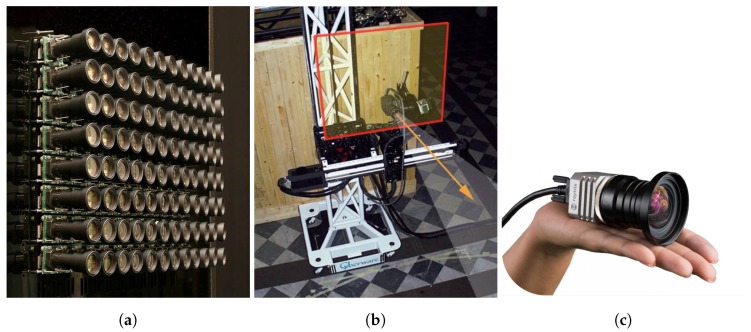
Examples of different implementations of light-field capturing devices: (**a**) camera rig mounting an array of eight by twelve cameras; (**b**) moving gantry shifting the camera in the area indicated by the red square; (**c**) plenoptic camera by Raytrix (image from https://raytrix.de/).

Camera rigs have been naturally constructed to capture the set of views, offering a high spatial resolution for each view but a low angular resolution (i.e., large baseline) [8]. Targeted applications include long range depth estimation, and augmented or virtual reality with immersive content. Single cameras mounted on moving gantries capturing the scene at regular time intervals have also been considered [9]. While camera rigs can be quite bulky and not easy to use, moving gantries are limited to capturing light fields of static scenes. Plenoptic cameras have also emerged based on novel optical designs [10,11]. Plenoptic cameras, thanks to an array of microlenses placed in front of the sensor, can be seen as multiplexing multiple low-resolution views in one 2D image sensor [11,12]. This is an efficient and easy way of capturing multiple viewpoints, even if the angular resolution is achieved at the expense of a decreased spatial resolution, when compared with classical 2D cameras. Despite the small baseline they offer (small disparities between views), they turn out to be low-cost and easy-to-use devices for light-field captures, and therefore for 3D scene reconstruction. Figure 3 illustrates some examples of the devices described above.

#### 2.1.1. Epipolar Plane Images

The concept of epipolar plane image (EPI) has been firstly introduced by Bolles et al. in 1987 [13], for building a three-dimensional description of a static scene from a dense sequence of images. The idea is derived from the epipolar constraint in stereo vision.

An EPI represents a spatio-angular 2D slice of the 4D light field, cut through a horizontal or vertical stack of light-field views (e.g., a (x,u) slice corresponding to the horizontal red line in Figure 2). It is obtained by fixing one of the spatial coordinates (e.g., by fixing the spatial coordinate *y* in Figure 2) and one of the angular coordinates (*v* in Figure 2). The EPI shown in Figure 2 therefore, gives an observation of a point at varying sub-apertures of the main lens or view positions *u* and at a given pixel location *x*.

Each observed 3D point, when projected in the EPI representation, traces lines with a certain slope that depends on the distance of the subject to the camera. The closer the camera is to the observed point, the steeper the slope is. This feature, as explained in the following paragraph, allows scene depth estimation.

#### 2.1.2. Scene Depth from Light Fields

The rich scene description provided by light fields enables 3D scene geometry estimation and 3D scene reconstruction. Scene depth estimation methods from light fields can be broadly classified in two categories, depending on the light-field baseline (large or small disparities between views). A first category of methods follows the principles of stereo matching using robust patch-based block matching [14,15,16]. A second category analyses specific linear structures in EPIs [17,18] for depth computation from dense light fields. In fact, the pixels corresponding to the same 3D point in different views of a light-field image form a line in the EPI whose slope is proportional to the disparity value [19]. Hereafter, those lines will be referred to as level lines. Please note that while stereo methods allow estimating larger disparities, EPI-based methods are only suitable for densely sampled light fields. This is the case for the light-field images treated by the works reviewed in this article, since they have been mostly captured by Lytro ILLUM plenoptic cameras.

#### 2.1.3. Refocusing from Light Fields

Densely sampled light fields make it possible to render images with shallow depth of field while controlling the focus depth. This set of images with different focusing depths is called the focal stack. Refocusing consists of defining a new light-field image $L^{\prime }\left(x,y,u,v\right)=L\left(x-us,y-vs,u,v\right)$, where *s* is the refocus parameter defined in such a way that the regions of disparity d=s in the light field *L* have zero disparity in the light field $L^{\prime }$. A refocused image $I^{s}$ with parameter *s* is computed by integrating the light rays over the angular dimension as [11]:

(1) $$I^{s}\left(x,y\right)=\int \int_{R}L\left(x-us,y-vs,u,v\right)\psi \left(u,v\right)dudv.$$

where ψ(u,v) represents the aperture of the imaging system, equal to 1 in the case of a full aperture. Therefore, refocusing is conceptually just a summation of shifted versions of the sub-aperture images over the entire ψ(u,v) aperture.

### 2.2. Face Analysis

Light-field images, acquired with a plenoptic camera or with an array of conventional 2D cameras, include information about different viewpoints of the scene. This allows developing face analysis algorithms that exploit the multiple viewpoints to achieve improved performance. These algorithms can explore the light-field characteristics, including functionalities such as:

- *A posteriori* refocusing—As mentioned in Section 2.1, it is possible to refocus a posteriori the captured image at a given depth plane, thus rendering a 2D image where the objects at the selected plane appear in focus. This functionality is not available when using a conventional 2D camera and it can be very useful for face analysis algorithms, allowing improvement of the analysis of a previously out-of-focus region of interest.
- Disparity exploitation—Given a captured light-field image, it is possible to render a set of 2D images corresponding to a set of specified viewpoints. This provides disparity information, with the differences between corresponding points/face components in different viewpoints providing valuable information for face analysis.
- Depth exploitation—As mentioned in Section 2.1, it is possible to estimate depth information from the light-field image. Knowing the distance between face components and the camera, it provides information about the scene geometry, useful for face analysis. An obvious usage of depth information is for presentation attack detection, where depth information can be used to determine if an image is captured from a flat surface (e.g., a screen) or not.

When working with light-field images it is possible to render 2D images that best describe the desired contents and then use traditional face analysis techniques. For instance, it is possible to use the refocusing functionality to create an all-in-focus 2D image, thus improving the analysis of faces that were captured at different focusing depths. Also, even if in principle depth and disparity express the same information, it can be useful to explore both types of information since different algorithms are used for their estimation. The alternative is to develop methods that directly explore the multidimensional light-field image, for instance, by processing the captured information as a tensor or as a multi-view array of 2D rendered images. The present paper overviews solutions of both types for a selected set of face analysis tasks.

The applications of face analysis are not limited to face recognition but also include other tasks such as face presentation attack detection (see Section 5), soft-biometrics categorization, and facial expression/emotion identification. The latter is the study of face features aiming to recognize the expression of the human face. Although this is a very studied field when dealing with traditional 2D cameras or other RGB-D or 3D sensors, until now, no works dealing with light field for human expression/emotion recognition have been published. Existing studies, employing other sensors, are mainly based on the extraction of face features and in the analysis of their changes when the face takes an expression. For an insight on recent techniques for facial expression recognition, the reader is referred to the survey paper by Corneanu et al. [19]. The present paper includes the review of a work dealing with face landmark localization on light-field images (see Section 3) that is an intermediary step for many face analysis applications, including face expression recognition.

Regarding soft-biometrics categorization, only one preliminary analysis work has been published so far. In [20], the authors observe a linear correlation between the depth of focus of the same face image, captured with a light-field camera and rendered at different focusing levels, and the gender and age scores obtained from it. Demonstrating the importance of controlling the image focus for this face analysis task.

As mentioned before, it is possible to exploit the rich information captured by light-field cameras to estimate depth and reconstruct 3D scenes. This feature, although not yet widely investigated, could be used to adapt and develop novel 3D face analysis techniques for light fields. A first step in this direction is described in [21], where the authors have compared the performances of a set of state-of-art algorithms for 2D and 3D face recognition on face images collected with two RGB-D sensors, which are a light-field camera and the Microsoft Kinect V1 (Read more at https://developer.microsoft.com/en-us/windows/kinect). Regarding the 3D information, the Kinect embeds a structured light 3D scanner. The 3D information is collected by projecting a known light pattern on the scene and analyzing how this pattern is deformed after hitting a surface. The results presented in [21], show that light fields are more robust than Kinect when dealing with facial occlusions (e.g., sunglasses). For a recent overview of 3D face recognition, the reader is referred to [22,23].

### 2.3. Databases

When discussing face analysis techniques, it is important to know which datasets were considered for their design, testing, and validation. By using publicly available databases it is possible to ensure that when face analysis solutions are reimplemented they achieve the initially reported performance results, while also guaranteeing the conditions for fair comparison among different techniques. Since this paper focuses on light-field face analysis, a summary of the existing light-field face databases is provided. These databases have been grouped into three categories:

- General-purpose light-field databases, which include facial images—These are databases initially developed with different purposes, but also happen to include face images. Metadata about the faces is typically not available, but depending on the purpose of the algorithms being developed, these face images might be interesting to consider. A summary of the available databases is listed in Table 1.
- Light-field face databases—These are databases developed specifically to test face recognition solutions. Therefore, they typically include metadata information, such as the face bounding box and facial component coordinates, the subject gender, age and appearance (facial hair, makeup, haircut, earrings, necklace, scarf, piercings, scars, etc.). There are also databases that focus on specific facial components, such as the iris or the ear, which were derived from light-field face databases, and are also listed in Table 2.
- Light-field face presentation attack detection databases—These databases were specifically developed to test face presentation attack detection solutions. Several types of presentation attack instruments have been considered, such as printed paper, laptop, tablet, and smartphone. Images acquired with a light-field camera of the presentation attack instruments are then tested to check whether the light-field information is helpful in distinguishing a presentation attack from a genuine user presentation (*bona fide*). The available databases for this category are listed in Table 3.

Most of the listed databases are made available for research purposes. Other private datasets have been used in some works, such as [24], but they are not listed as they are not available for the research community. More detailed information about the content of each of the listed databases can be found in the provided references.

## 3. Face Landmark Detection

Face landmark detection is the process of detecting salient points of the face, often corresponding to the edges of the main components of the face (e.g., mouth and eye corners, nose tip, chin, etc.). This is an intermediary step for many face-processing applications, including face recognition, face morphing, face liveness detection, and presentation attack detection. Recent research adopts face landmarks for face expression or emotion recognition by analyzing the dynamics of the landmarks while the face changes expression. Among more recreational applications, face landmarks are used on smartphones to superimpose, with surprising accuracy, funny masks or makeup or to apply “beauty filters” that operate differently on different areas of the face and thus require accurate face landmark detection.

**Figure 4:**
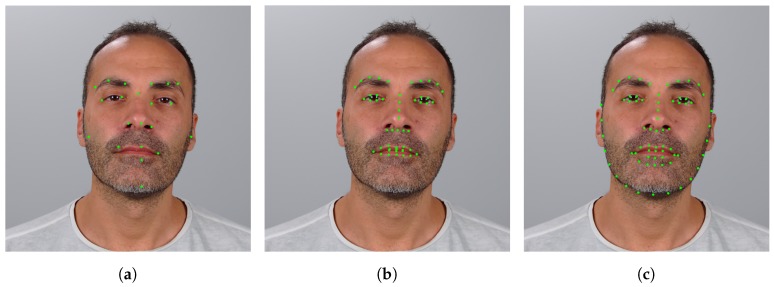
Face landmark detection: an example of output of three face landmark detectors. Face image extracted from the IST-EURECOM Light-Field Face Database (LFFD). (**a**) CLandmarks [40]; (**b**) IntraFace [41]; (**c**) DLIB.

Face landmark detection has proven extremely challenging due to the inherent face variability [38,39]. While achieving high accuracy on standard face images (e.g., frontal pose, neutral expression, standard illumination), state-of-the-art methods still suffer a significant drop in performance when dealing with different factors including pose, expression, illumination, and occlusions. An example of face landmark detection performed by different algorithms is presented in Figure 4.

**Figure 5:**
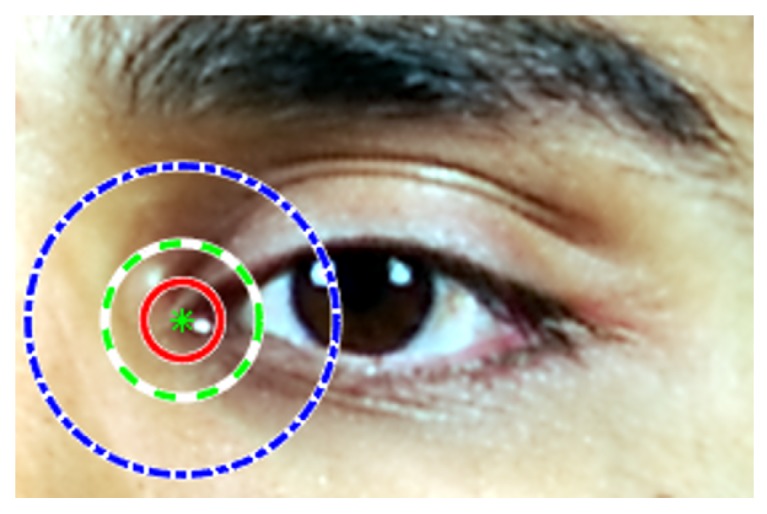
Detection thresholds of the eye inner corner. Concentric circles denote error ranges with radii 0.05 (red solid line), 0.1 (green dashed line), and 0.2 (blue dash-dotted line) times IOD, respectively. Eye image extracted from the IST-EURECOM LFFD.

To evaluate the performance of a face landmark detector, two different approaches can be defined: (i) compare the estimated landmarks with the ground truth, (ii) evaluate the performance for a specific task (e.g., emotion recognition). A straightforward way to assess landmark localization performance is to use manually annotated ground truths. A largely used metric is the normalized root mean square error (NRMSE). The normalization is typically done with respect to inter-ocular distance (IOD), which is defined as the distance between the two eye centers. Normalizing landmark localization errors by dividing by IOD makes the performance measure independent of the actual face size or the camera zoom factor [38]. The NRMSE between the ground-truth coordinates (x,y) and the estimated coordinates $\left(\tilde{x},\tilde{y}\right)$, is defined as:

(2) $$\delta_{i}^{k}=\frac{d\{\left(x_{i}^{k},y_{i}^{k}\right),\left({\tilde{x}}_{i}^{k},{\tilde{y}}_{i}^{k}\right)\}}{IOD}$$

where d() indicates the Euclidean distance, *k* indicates the landmark index (e.g., eye corner, nose tip) and *i* is the image index. The overall landmark detector performance in terms of percentage of detected landmarks *P*, is computed by the following formula:

(3) $$P=100\frac{\sum_{k=1}^{K}\sum_{i=1}^{N}\left[i:\delta_{i}^{k}<Th\right]}{K\times N}$$

where $\left[i:\delta_{i}^{k}<Th\right]$ is the indicator function of value 1 if the distance is smaller than Th, otherwise its value is 0. *N* denotes the number of test images and *K* the number of landmarks per face image. The threshold Th defines the error tolerance of the metric. The landmark estimation errors are assumed isotropic, so that one can conceive around each ground-truth landmark a detection circle with radius equal to the error threshold. The example in Figure 5 illustrates different error ranges corresponding to different Th values. The detection threshold corresponds to a percentage of the IOD, and it is typically set to 10%, or below, of IOD (i.e., Th<=0.1).

The rich information provided by light-field data has pushed the researchers to consider how face landmark detection could be adapted to this new paradigm.

### 3.1. Facial-Landmark Localization Correction

**Figure 6:**
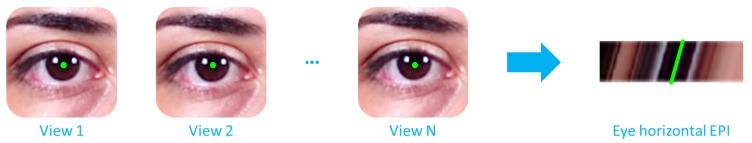
Level line: a 3D point lies on a straight line in the epipolar plane image. Eye image extracted from the IST-EURECOM LFFD.

The work presented in [42], explores the observation that in the EPI—described in Section 2.1—a 3D point is represented by a straight line. Thus, the position estimation of a face landmark on a light-field image can be optimized by forcing the estimated points to be on a straight line. Hereafter, such lines will be referred to as level lines, see Figure 6.

The idea behind the work presented in [42] is thus to detect the EPI’s level lines and use them to correct landmark position estimation to improve localization accuracy in terms of NRMSE.

One way to detect the EPI’s level lines, is to use structure tensors. The structure tensor, also referred to as the second-moment matrix, is derived from the gradient of the EPI as:

(4) $$J_{\sigma }\left(x,u\right)=\Delta E_{y^{*}}{,}_{v^{*}}\left(x,u\right)\cdot \Delta E_{y^{*}}{,}_{v^{*}}{\left(x,u\right)}^{T}*G_{\sigma }$$

where $G_{\sigma }$ is a Gaussian smoothing operator of variance $\sigma^{2}$. The orthogonal eigenvectors $V_{+}$ and $V_{-}$ with respective eigenvalues $\lambda_{+}$ and $\lambda_{-}$ (where $\lambda_{+}>\lambda_{-}$) of J(x,u) give a robust computation of the local gradient orientations locally at (x,u). The eigenvector $V_{-}$ with the smallest eigenvalue describes the director vector of the level lines passing through (x,u).

#### 3.1.1. Coordinates Correction

**Figure 7:**
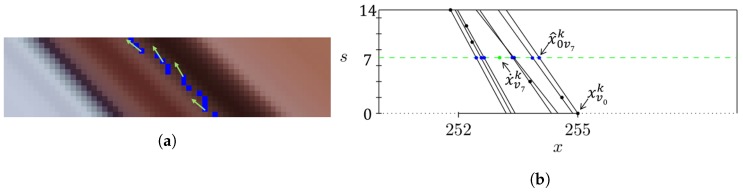
Coordinates correction: in (**a**), the scattered line produced by an estimated landmark point over the horizontal views; in (**b**), magnified view of the level lines computed using structure tensors.

An ideal face landmark detector would localize the landmarks with perfect precision over all the views of the same light-field face image. This would create straight lines on the EPI, corresponding to the face landmarks. It is observed instead that even if the sub-aperture images are extremely similar—they only differ of a little horizontal and vertical disparity from each other—the landmark position estimation is different from view to view. This is even more evident on more challenging faces showing different expressions or pose. In the latter case, the estimated points would be scattered around the ground-truth level line. See the example given in Figure 7a.

The level lines corresponding to a landmark point *k* detected on the *N* views are computed using structure tensors and used to correct the coordinates of the landmark on the central view (i.e., view with index N/2).

An example of *x* coordinate correction is illustrated in Figure 7b. The same process is then applied to *y* coordinates. Please note that in the following formula and in Figure 7b the naming convention used in [42] is adopted: (s,t) denote the angular or view coordinates. The corrected coordinate ${\dot{x}}_{v_{c}}^{k}$ of the $k^{th}$ landmark on the central view $v_{c}$, is obtained through a weighted sum of the projected points ${\hat{x}}_{s_{v_{c}}}^{k}$ that is the projection of the point $x_{v_{s}}^{k}$ from the view *s*, where s=0,1,2,…,P, on the central view (blue points in Figure 7b):

(5) $${\dot{x}}_{v_{c}}^{k}=\frac{\sum_{s=0}^{P}w_{v_{s}}^{k}\cdot {\hat{x}}_{s_{v_{c}}}^{k}}{\sum_{s=0}^{P}w_{v_{s}}^{k}}.$$

The weights are used to give more importance to points that are close to each other and less importance to eventual outliers. The weight $w_{v_{s}}^{k}$ is defined as the number of x-coordinates that are at a distance less than 0.1 pixel from the level line of the corresponding point $x_{v_{s}}^{k}$.

#### 3.1.2. Results

Performance is assessed in terms of percentage of detected landmarks *P* on a set of 400 face images from the IST-EURECOM Light-Field Face Database (LFFD) database, including different face variations. Face landmarks are initially detected by DLIB (Read more about DLIB at http://blog.dlib.net/), a C++ library by Davis King that implements the method presented in [43] for face alignment based on regression trees. The results presented in Table 4 show that the method is particularly effective on more complex face variations, due to less accurate landmark localization of DLIB on these kinds of images. As a result, the correction is more beneficial.

## 4. Face Recognition

Automatic face recognition is becoming increasingly used for automated border control (ABC), access to protected areas or even to unlock smartphones. A wide literature on face recognition has been developed in recent decades, providing algorithms able to deal with challenging acquisition environments or with low-resolution images. With the improvement of optics, different sensors have been conceived, from time-of-flight cameras to light-field devices.

The works reviewed in this section adopt a different terminology when reporting performance. To provide a common understanding of the reported metrics, a standard terminology is used here, derived from the ISO/IEC 19795-1 [44]. Although accuracy is not a standard metric, we report it here as it is used in one of the reviewed works. Performance is reported in terms of the following metrics:

- **IR**: Identification Rate at rank 1–The (true-positive) identification rate at rank 1 is the proportion of identification transactions by a user enrolled in the system, for which user’s true identifier is returned in first position in the candidate list. If the rank is omitted in the following, rank 1 is implied.
- **EER**: Equal Error Rate—value corresponding to FMR=FNMR
  – **FMR**: False Match Rate—proportion of the completed biometric non-mated comparison trials that result in a false match;
  – **FNMR**: False Non-Match Rate—proportion of the completed biometric mated comparison trials that result in a false non-match.
- **ACC**: Accuracy—corresponding to the average value of TMR and TNMR;
  – **TMR**: True Match Rate—proportion of the completed biometric mated comparison trials that result in a true match;
  – **TNMR**: True Non-Match Rate—proportion of the completed biometric non-mated comparison trials that result in a true non-match.

**Figure 8:**
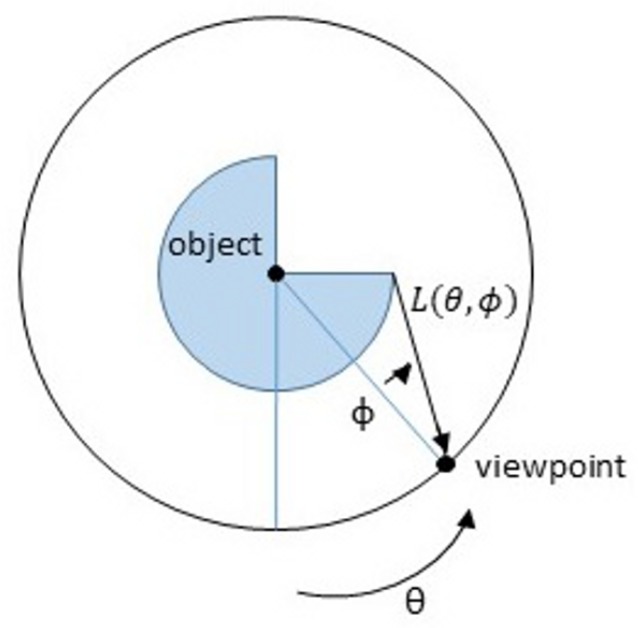
A visual description of the method used to define light field from 2D images in [1,2,4]. The object is conceptually placed within a circle. The angle to the viewpoint around the circle is measured by the angle θ, and the direction that the viewing ray makes with the radius of the circle is denoted ϕ. For each pair of angles θ and ϕ, the radiance of light reaching the viewpoint from the object is then denoted by L(θ;ϕ), the light field.

While most of the algorithms proposed for light-field face analysis are customized to be applied on data acquired with plenoptic cameras (Lytro devices most often), some works presented before 2006 use as database images collected with conventional sensors. In [1,2,4], Gross et al. suggest exploiting pose and illumination variations collected in the CMU PIE [45] and FERET databases [46] to create a light-field model of the face. Given a 2D image, the pixels belonging to the face are detected and the corresponding light-field image is computed as shown in Figure 8.

The same concept was explored in 2011 by Wibowo et al. [47] to perform face recognition from video sequences.

(6) $$I-\sum_{i=1}^{d}\lambda_{i}W_{i}\left(\theta ,\phi \right)=0$$

In [3], Zhou et al. integrate the Lambertian reflectance model to the method proposed by Gross et al. to consider illumination variations in addition to different poses.

The surveyed works in the following Subsections, employ light-field images collected with actual plenoptic cameras. They are organized in three categories depending on the image representation exploited. The categories are the following: Multi-focus-based methods; Sub-aperture-based methods, and deep-learning algorithms.

### 4.1. Multi-Focus Based Methods

**Figure 9:**
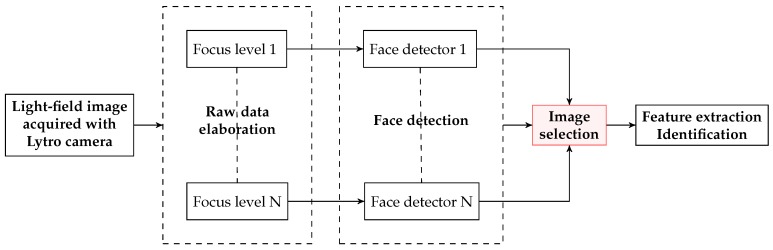
Workflow proposed in [5].

**Figure 10:**
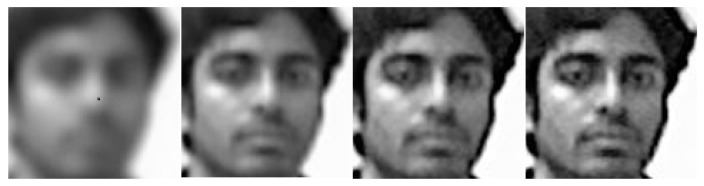
An example of image rendered at different focus levels: The pictures are all extracted from the same light-field image. Image from the GUCLF database.

The first studies on face recognition on light-field images collected by plenoptic cameras, are based on the use of 2D images rendered from the light-field image at different focusing depths. In 2013, Raghavendra et al. [5] published an innovative technique to extract the best focused images from a set of 2D images from different depth planes. The authors presented one of the first light-field databases for face recognition [30] and proposed an approach to detect, select and extract features from light-field images (Figure 9). The main steps of the method are summarized below:

- The 2D images in the database are obtained by rendering the original light-field files at different focusing depths (Figure 10). All images are then processed with the Viola-Jones face detector [48] trained with 2429 face images and 3000 non-face samples. For each capture, the rendered image where the largest number of faces is detected, is chosen to define the facial regions.
- Once the faces are detected and cropped, the best image for each individual is selected according to an energy criterion. The authors chose as energy measure the 2D-Discrete Wavelet Transform (DWT) with Haar wavelet because of its robustness to noise and its content-independent property. The face image with larger energy is chosen.
- Local Binary Pattern (LBP) features [49] and Log-Gabor (LG) features [50] are extracted from the selected image. They are then used as input of Kernel Discriminant Analysis (KDA) [51] and Sparse Reconstruction Classifier (SRC) [52].

During the creation of the database, the images were acquired with both light-field and conventional cameras. The purpose of the work presented in [5] is to compare the identification rate obtained on light fields with the one achieved on standard 2D-images. The results show an increase in performance of 7.15% identification rate when using different state-of-the-art algorithm separately. The best-performing algorithm for 2D images is LG-SRC (53.97% IR), while for light field is LBP-SRC (61.12% IR). However, when the algorithms are fused at decision level, the improvement is only of 3.57%. Table 5 reports the obtained results.

In [53], the same authors investigate the use of light fields for identifying multiple faces present at different distance. From the GUCLF database, the depth information is exploited by combining the multi-focus images according to two strategies: to obtain an all-in-focus image; to obtain a super-resolution image. In this preliminary work, state-of-the-art super-resolution techniques [54,55,56,57,58] are used. The best performance is achieved using all-in-focus images and in the outdoor scenario with an identification rate at rank 1 of 53.62%. The best-performing super-resolution scheme, only achieves 32.86% identification rate. However, the authors do not compare the proposed methods with a 2D-image baseline.

A novel weighted image fusion scheme is proposed by Raghavendra et al. in [59]. While the face detection remains unchanged compared to the first work [5], image selection is based on entropy. For each focus version of each detected face, 2D-DWT is applied and the log-entropy is assessed with the following equation:

(7) $$E=-\sum_{i=1}^{K}\left(log_{2}{\left(W_{i}\right)}^{2}\right)$$

where $W_{i}$ for $i\in \left(1:K\right)$ are the resulting wavelet coefficients.

Only samples with positive entropy are kept. The image entropy is normalized and sorted in decreasing order so that the best-in-focus image appears as first. The difference between adjacent entropy values (see Equation (8)) is used to assign a weight to each image.

(8) $$D_{j}=\left|Sor_{j+1}-Sor_{j}\right|\;for\;j\in \left\{1:\#images\right\}$$

(9) $$w_{j}=\left\{\begin{array}{c} \left(0.5+\left(0.5*D_{j}\right)\right)*Max_{w}\;\;if\;D_{j}\geq Th\\ \frac{Max_{w}}{2}\;\;\;\;otherwise \end{array}\right.$$

where Th is empirically set to 0.2 and $Max_{w}$ is initially equal to 1 and then updated for each sample *j* with the weight value of the sample j+1.

The image samples are then fused with the weighted sum rule (see Equation (10)).

(10) $$W_{f}=\sum_{j}W_{j}*w_{j}$$

where $W_{j}$ is the $j^{th}$ image in the discrete wavelet domain and $w_{j}$ the corresponding weight. The final result $W_{f}$ is converted in spatial domain and used to extract features to perform face recognition.

The results obtained by using the proposed weighted image fusion scheme, are compared with the performance obtained by only selecting the image with largest entropy. The identification rate achieved with the fusion scheme is higher in all the considered scenarios. The best result is achieved when LBP-SRC and LG-SRC are fused at decision level using the OR rule. The identification rate is of 75.12%. The proposed method is not compared with any 2D-image baseline.

The scheme is further improved in [60] where a new hybrid resolution enhancement technique is proposed. Also in this case, the first step of face detection remains unvaried. As in [59], 2D-DWT is performed on the images by applying filtering and downsampling, with high-pass (H) and low-pass (L) filters over rows and columns. This process produces four sub-images: $I_{LL}$, $I_{LH}$, $I_{HL}$ and $I_{HH}$. For each sub-image, the wavelet energy is calculated and the image with largest energy is selected to represent the sample. The sub-image $I_{LL}$, containing the lower-frequency band, is replaced by a super-resoluted version of the original image obtained with a state-of-the-art method [54,55,56,57,58]. The obtained results show that the algorithm outperforms other well-known super-resolution techniques in terms of identification rate: 60.56% (Indoor), 58.87% (Corridor) and 51.47% (Outdoor).

In 2015, Raja et al. [61] studied the problem of light-field depth image fusion and in particular how to optimize the number of images to be fused. Following an approach inspired by [59], they consider in the fusion scheme only the two images with highest energy. As for [60], the energy is calculated from the energy sum of three sub-images obtained with low-pass and high-pass filtering. The best equal error rate of 4.14% is obtained with the combination of the proposed selection method and Laplacian Pyramid-based fusion using both sparse representation and multi-scale transform.

Table 5 reports the performance of the methods analyzed in this section.

### 4.2. Sub-Aperture Based Methods

The publication of the IST-EURECOM LFFD [32] in 2017, has paved the way for the development of approaches based on the full information provided by light-field images. For example, the access to raw data allows investigation of the impact of sub-aperture representation on face recognition. So far, two main works have been proposed using the LFFD database, with the aim of improving face identification rate over standard algorithms based on 2D face images.

**Figure 11:**
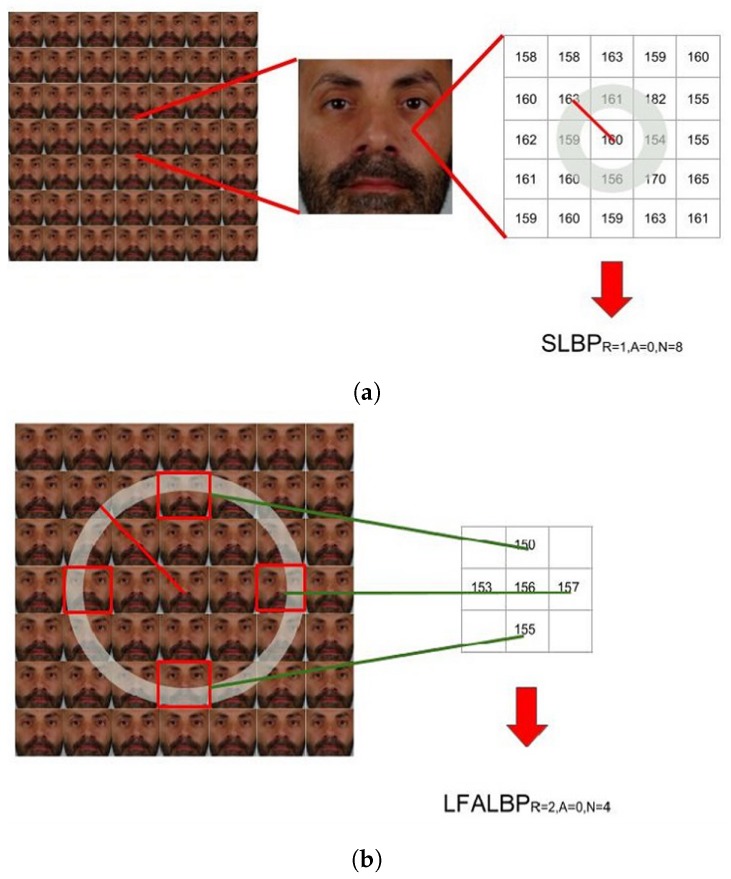
Visual representation of (**a**) Spatial Local Binary Pattern (SLBP) and (**b**) Light-Field Angular Local Binary Pattern (LFALBP) used in [62].

The first method is proposed by Sepas-Moghaddam et al. [62] and is inspired by LBP [49]. While for the computation of the classic LBP feature vector, adjacent pixels are considered, the Light-Field Local Binary Pattern (LFLBP) is composed by an additional component that includes the information stored in adjacent views (Figure 11):

- Spatial Local Binary Pattern (SLBP): the first component is the LBP feature vector extracted from the central view of the considered light-field image;
- Light-Field Angular Local Binary Pattern (LFALBP): the second component is a variation of classical LBP customized for light-field images. Let (x,y) be the reference sample and *R* the radius representing the distance of the selected adjacent views from the central view. Then, the LFALBP is defined as:
  (11) $$LFALBP_{R,A,N}\left(x,y\right)=\sum_{j=1}^{N}sign\left(L_{u,v,x,y}-L_{0,0,x,y}\right)*2^{j-1}$$
  where
  (12) $$\left\{\begin{array}{c} u=\left[Rsin\left(A+\frac{360^{{}^{\circ}}}{N}*\left(j-1\right)\right)\right]\\ v=\left[Rcos\left(A+\frac{360^{{}^{\circ}}}{N}*\left(j-1\right)\right)\right] \end{array}\right.$$
  Angle *A* indicates the starting angle for the first sub-aperture image to consider in the angular neighborhood, and *N* indicates the number of views to consider. As in the conventional LBP descriptor, the binary thresholding result obtained by the sign function is multiplied by the binomial factor, $2^{j-1}$, and the resulting values are summed to get the LFALBP pattern value for each sample position (x,y).

The authors have tested the proposed LFALBP with different sets of parameters and compared the proposed approach with several state-of-the-art techniques. The average results over three recognition tasks is of 92.1% identification rate, outperforming the best-performing 2D-image-based state-of-the-art method of 3%.

**Figure 12:**
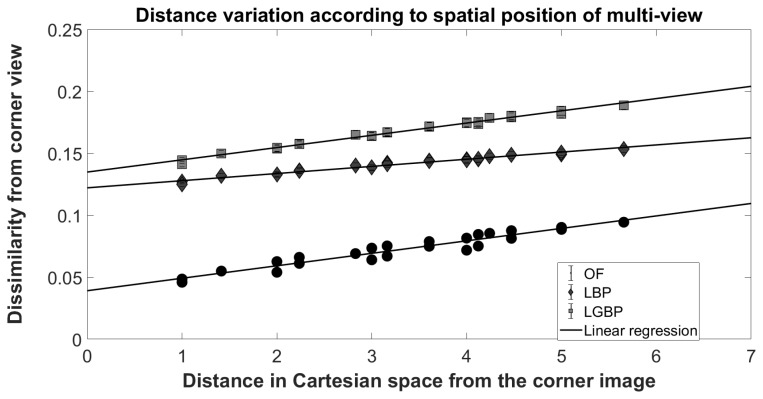
Relation between the distance of a pair of views in Cartesian space and the face recognition dissimilarity score: The observed linear correlation proves the feature extractor capability to capture the small face variation present in each view.

The second work is presented in [63]. As for [62], the authors exploit the sub-aperture representation of light-field face images. For each view of a light-field image, OpenFace (OF) [64], LBP [49] and LGBP [65] features are extracted. The impact of perspective shifting is demonstrated showing a linear relation between view shifting and face recognition dissimilarity score (see Figure 12).

To evaluate the similarity of two light-field face images, all feature vectors extracted from the views of one image are matched with all feature vectors extracted from the other one. As comparison score, the Euclidean distance $L^{2}$ distance for OF features and chi-squared $\chi^{2}$ distance for LBP and LGBP are used. Eight pseudo-distances are defined to combine the multiple values, obtained by comparing the features from the different views, to a single one.

Let *A* and *B* be feature vector sets describing the first and the second image, respectively, and $A_{C}$ and $B_{C}$ the feature vectors obtained from the corner view. The pseudo-distances are defined as:

- $d_{min}=min_{a\in A,b\in B}\left(d_{s}\left(a,b\right)\right)$
- $d_{minC}=min_{a\in A_{C},b\in B_{C}}\left(d_{s}\left(a,b\right)\right)$
- $d_{mean}=mean\left(d_{s}\left(a,b\right)\;\forall a\in A,\forall b\in B\right)$
- $d_{meanC}=mean\left(d_{s}\left(a,b\right)\;\forall a\in A_{C},\forall b\in B_{C}\right)$
- $d_{max}=max_{a\in A,b\in B}\left(d_{s}\left(a,b\right)\right)$
- $d_{maxC}=max_{a\in A_{C},b\in B_{C}}\left(d_{s}\left(a,b\right)\right)$
- $d_{H_{mean}}=\frac{1}{\#A+\#B}\left(\sum_{a\in A}min_{b\in B}\left(d_{s}\left(a,b\right)\right)+\sum_{b\in B}min_{a\in A}\left(d_{s}\left(a,b\right)\right)\right)$
- $d_{H_{max}}=max\left(max_{a\in A}\left(min_{b\in B}\left(d_{s}\left(a,b\right)\right)\right),max_{b\in B}\left(min_{a\in A}\left(d_{s}\left(a,b\right)\right)\right)\right)$

The evaluation is conducted by comparing the results obtained by the proposed method with the results of standard 2D-image-based algorithms applied on the central view. Even though the baseline performances are already really good (EER lower than 0.05% when OF features are used), the authors prove that by using light field richer information it is possible to increase the performances by 0.53%.

Table 6 summarizes the performances of the methods described in this section.

### 4.3. Deep-Learning Algorithms

In 2018, Sepas-Moghaddam et al. used for the first time a deep-learning approach on light-field images to deal with the face recognition [66]. In this work, the authors fuse several representations of the raw data to exploit as much as possible the information stored in the image. Features are extracted by three VGG-Face neural networks [67] fused to feed a Support Vector Machine classifier (SVM). The main steps of the proposed approach are summarized below:

- The central view is the input of a pre-trained VGG-Face model;
- The disparity map calculated from the sub-aperture representation is used to fine-tune a second VGG-Face model;
- A third VGG-Face model is fine-tuned using depth maps.

**Figure 13:**
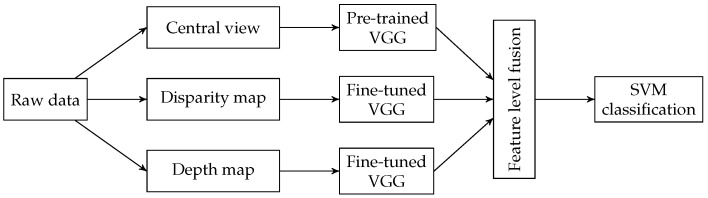
Workflow of the method proposed in [66].

Figure 13 illustrates the workflow of the method. The analysis of the rank-1 identification rate shows that the proposed algorithm, when fusing all available information, i.e., 2D+disparity+depth, achieves 98.1% identification rate. An improvement of 1.3% when compared to the use of 2D-image information only.

The same authors, in a second work, have developed a double-deep spatio-angular learning structure [68] based on the analysis of several images obtained from the sub-aperture representation. Each considered view is processed with a pre-trained VGG-Face network to extract a 4096-dimensional spatial feature vector. The output of this first elaboration is used as input for a Long Short-Term Memory (LSTM) Recurrent Neural Network [69] that extracts the angular dependencies across the views. The last step is a SoftMax classifier able to indicate the most probable identity of the individual represented in the image.

**Figure 14:**
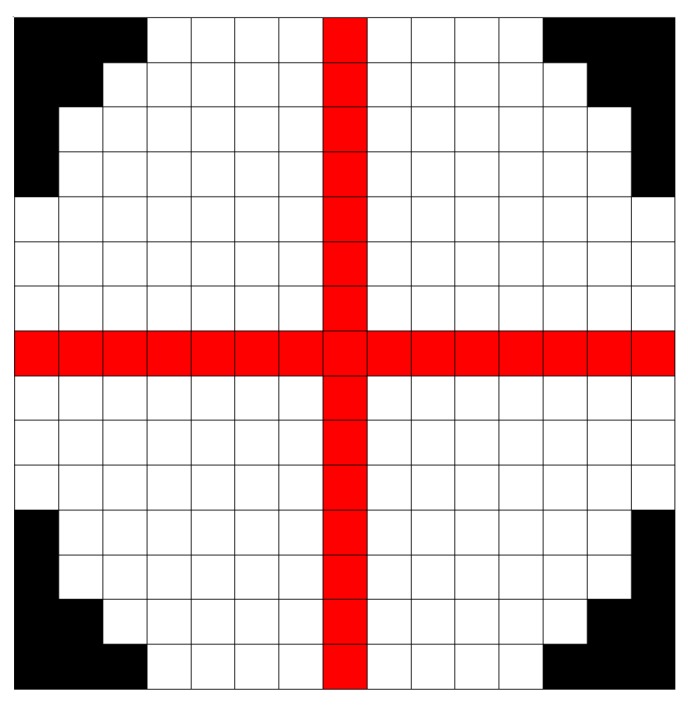
View selection scheme proposed in [68]: The red squares represent the position of the views selected in the mid-density horizontal and vertical configuration.

The use of all views extracted from a light-field image would be computationally expensive and not necessary. The authors compare the application of this method on different set of views, selected based on different schemes. For example, the configuration called “Mid-density horizontal and vertical” is shown in Figure 14, where the selected views are those highlighted in red. The achieved identification rate is 98.60%, improving the performance of their previously proposed method of 1.2%, and of 5.7% when compared with the best-performing 2D-image-based approach.

Table 7 reports the performances achieved by the methods described above.

## 5. Face Presentation Attack Detection

Besides the already mentioned airport application of ABC gates, access to mobile banking applications through face recognition, which by nature of the private interaction is unsupervised, motivates the importance of presentation attack detection. Over the years, academic and industry research centers developed countermeasures to detect biometric presentation attacks.

In general, a presentation attack can be conducted from an attacker with limited skills that interacts with a face capture device. The scenario of such attacks is well defined in the international standard that is entitled “ISO/IEC Information Technology–Biometric presentation attack detection” [70]. The intention of this standard is to provide a harmonized definition of terms and a taxonomy of attack techniques and a testing methodology that can evaluate PAD mechanisms.

### 5.1. Taxonomy for Presentation Attack Detection

In a multi-disciplinary community as in biometrics there is a tendency to struggle with a clear and non-contradictory use and understanding of its terms. Thus, ISO/IEC has undertaken significant efforts to develop a Harmonized Biometric Vocabulary (HBV) [71]. To formulate a common understanding of attacks on biometric systems the HBV was expanded with the following concepts that are provided in ISO/IEC 30107-1 Biometric presentation attack detection–Part1: Framework [70] and in ISO/IEC 30107-3 Biometric presentation attack detection–Part3: Testing and reporting [72]. Here, some of the standard terms are reported, which will be useful for the understanding of the following Sections:

- **presentation attack/attack presentation**: presentation to the biometric data capture subsystem with the goal of interfering with the operation of the biometric system
- **bona fide presentation**: interaction of the biometric capture subject and the biometric data capture subsystem in the fashion intended by the policy of the biometric system
- **presentation attack instrument (PAI)**: biometric characteristic or object used in a presentation attack
- **PAI species**: class of presentation attack instruments created using a common production method and based on different biometric characteristics
- **artefact**: artificial object or representation presenting a copy of biometric characteristics or synthetic biometric patterns
- **presentation attack detection (PAD)**: automated determination of a presentation attack

The framework defined in [70] considers two types of attacks. On the one hand, the *Active Impostor Presentation Attack* is considered, which attempts to subvert the correct and intended policy of the biometric capture subsystem and in which the attacker aims to be recognized as a specific subject known to the system (e.g., an impersonation attack). On the other hand, the *Identity Concealer Presentation Attack* as attempt of the attacker to avoid being matched to its own biometric reference in the system.

An attacker be it an active impostor or an identity concealer will use an object for their attack that is interacting with the capture device. Moreover, the potential of their attack will depend on their knowledge and the window of opportunity. However, for the object that is employed we can anticipate for attacks against face capture devices as simple face image print outs or facial images being displayed on resolution tablets. Moreover, an attacker might present their genuine characteristic, but identification is avoided with non-conformant behavior with respect to the data capture regulations, e.g., by extreme facial expression or active visors.

### 5.2. Metrics for PAD Subsystem Evaluation

For a secure biometric system, the capture subsystem would be augmented with a PAD subsystem, which forwards the captured sample only then to the comparison subsystem, if it has been classified as bona fide. Such classification by the PAD subsystem is again subject to potential errors. Thus, when it comes to the testing of the detection subsystem ISO/IEC 30107-3 introduced metrics for PAD evaluation. A PAD subsystem shall be evaluated using two metrics namely [72]: (1) Attack Presentation Classification Error Rate (APCER): defined as the proportion of presentation attacks incorrectly classified as *Bona Fide* presentations (2) Bona Fide Presentation Classification Error Rate (BPCER): defined as the proportion of *Bona Fide* presentations incorrectly classified as presentation attacks.

The APCER can be calculated as follows:

(13) $$APCER=\frac{1}{N_{PAIS}}\sum_{i=1}^{N_{PAIS}}\left(1-RES_{i}\right)$$

where $N_{PAIS}$ is the number of attack presentations for the given PAI [70]. $RES_{i}$ takes the value 1 if the $i^{th}$ presentation is classified as an attack presentation and value 0 if classified as *Bona Fide* presentation.

While the BPCER can be calculated as follows:

(14) $$BPCER=\frac{\sum_{i=1}^{N_{BF}}RES_{i}}{N_{BF}}$$

where $N_{BF}$ is the number of *Bona Fide* presentations. $RES_{i}$ takes the value 1 if the $i^{th}$ presentation is classified as an attack presentation and value 0 if classified as *Bona Fide* presentation.

Some of the works presented in the following section report performance in terms of half total error rate of APCER and BPCER: $HTER=\frac{APCER+BPCER}{2}$. Please note that the HTER has been deprecated by the International Standard ISO/IEC 30107-3.

### 5.3. State-of-the-Art PAD with Light-Field Capture Devices

The vulnerability of face recognition capture devices for presentation attacks has intensively being discussed in the literature. For a recent overview, the reader is referred to [73], which also includes a discussion on countermeasures based on texture-based approaches or challenge response protocols. Given the low level of difficulty to render high-resolution video material on low-cost tablets, the limits to detect such attacks with a conventional 2D face capture device are obvious.

Soon after the light-field capture devices became available, they were investigated as means of defense against presentation attacks with 2D PAI. The motivation is straightforward as the light fields provide a superset of data acquired from the capture subject, which allows not only focus analysis at various depth and disparity exploitation.

**Figure 15:**
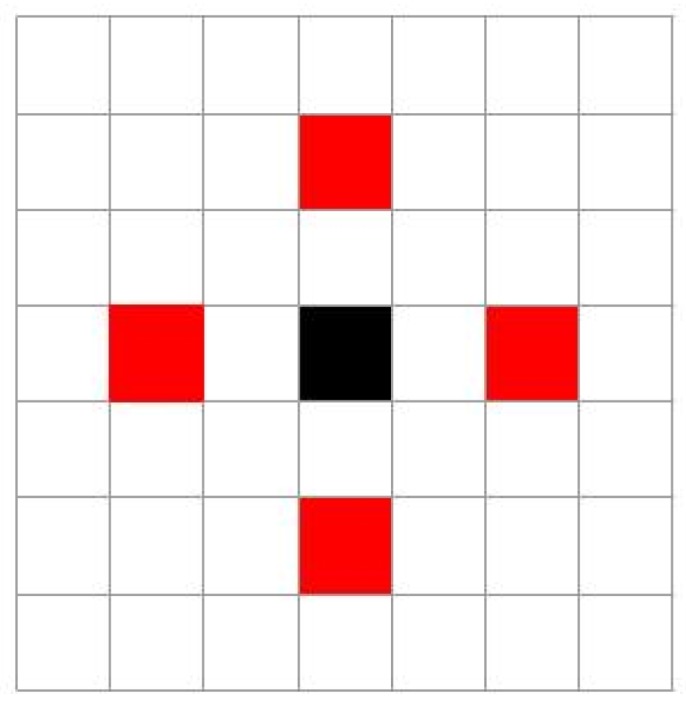
Map of views studied to evaluate the ray-difference features as described in [74].

Kim et al. [74] investigated two features to distinguish bona fide presentations from attack presentations conducted with printed PAD and high-resolution tablet. The first feature, namely the edge feature, is based on an extension of the LBP algorithm computed only on the areas located on the edge of the lower jaw. Whereas in [62] a similar LBP extension is proposed but the features are extracted from the sub-aperture representation, in [74] the inner and outer binary pattern methods are applied on microlens images. The microlens image is the raw information captured by the light-field camera sensor. It depicts the scene captured by each single microlens. This information is then processed to obtain the sub-aperture representation (see Figure 2). The authors define as inner binary pattern the LBP of average values evaluated for each microlens image. The outer binary pattern is computed on surrounding microlens images. The second feature is based on sub-aperture representation, and is called ray-difference image. The ray-difference image is computed on the whole face area and is obtained by computing the difference between the central view and 4 other views (see Figure 15) at a given distance from the central one. The output are 4 error maps where the areas with larger depth change have the larger difference. The classical LBP algorithm is applied on the resulting images and the features are concatenated to feed an SVM. The work reported a half total error rate of APCER and BPCER that is in the range of 0.89% to 4.10% for the edge feature and 2.5% to 4.22% for the ray-difference feature.

In Raghavendra et al. [34] the focus variation between images at multiple depths is analyzed. As for [5], the authors render each light field at different focusing depths and detect faces in the obtained 2D images. Two schemes are proposed: the first explores the variation in focus among images extracted from the same raw data; the second is based on a simple decision rule on the number of depth images rendered by the Lytro camera on a single capture. The employed PAIs are collected in a dedicated dataset, namely the GUC Light-Field Face Artefact Database (GUC-LiFFAD), and include either high quality printed facial photos (printed using both laser and ink jet printers) and an electronic display. Detection accuracy is reported with the half total error rate of APCER and BPCER that varies from 4.01% to 5.27% depending on the PAI.

Later, Ji et al. [75] proposed a PAD subsystem that is based on the Light-Field Histogram of Gradients (LFHOG) descriptor that extends the classical Histogram of Oriented Gradients (HOG) by considering gradients in three directions. While horizontal and vertical gradients are calculated as for standard HOG on the rendered 2D image, the gradient in depth direction is evaluated as the difference between two refocused images at different depths. Equation (15) describes the gradient in depth direction.

(15) $$G_{z}\left(x,y\right)=I_{s_{1}}\left(x,y\right)-I_{s_{2}}\left(x,y\right)$$

where $I_{s}\left(x,y\right)$ is described in Equation (1). $I_{s_{1}}$ and $I_{s_{2}}$ are the refocused images in the reference depths $s_{1}$ and $s_{2}$, respectively. The module *r* and orientations θ and ϕ (in this case the orientations are two since the gradient vector is 3 dimensional) of the gradient are defined as in Equation (16).

(16) $$\begin{array}{c} r=\sqrt{dx^{2}+dy^{2}+dz^{2}}\\ \theta =\arccos\frac{dz}{r}\\ \phi =\arctan\frac{dy}{dx} \end{array}$$

where (dx,dy,dz) are the components of the gradient of the light-field image in horizontal, vertical, and depth directions, respectively. The features are classified via SVM. The approach combines the analysis of the distribution of color intensity and of the distribution of scene depth. Detection accuracy is reported as 99.75% while omitting details on APCER and BPCER.

**Figure 16:**
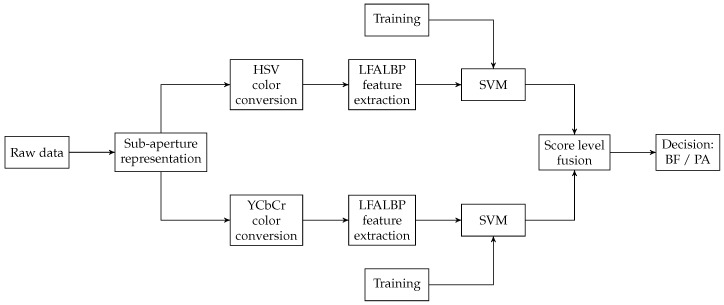
Schematic representation of the workflow for computing the $LBP_{HVS+YC_{b}C_{r}}$ features [35].

In 2018, Sepas-Moghaddam et al. [35,36] presented an overview of light-field-based PAD and developed novel methods exploiting the disparity information available in light-field images. The experiments are performed on the IST Lenslet Light-Field Face Spoofing Database (IST LLFFSD), the first face artefact database to include the raw light-field imaging files. In [35], they analyze color and texture variations associated with the different directions of light captured in light-field images. The algorithm is quite similar to the one for face recognition they previously presented in [62]: an extension of the classical LBP algorithm, customized for light-field images, is defined, namely the light-field angular LBP (LFALBP). Instead of considering adjacent pixels, values from adjacent views, transposed in the HVS and $YC_{b}C_{r}$ color spaces, are used. In this scheme, two classifiers are created, one trained with $LFALBP_{HVS}$ features and one with $LFALBP_{YC_{b}C_{r}}$. Scores are merged to give the final classification result. Figure 16 shows a schematic representation of the framework. The proposed solution achieved the best performance compared to both 2D and LF-based state-of-the-art solutions. The average HTER detection error obtained ranges between 0.33% and 2.85%.

The method presented in [36] is inspired by the work in [35] but, instead of the LBP algorithm, it extends the well-known HOG method [76]. The authors propose the so-called Histogram of Disparity Gradients (HDG) descriptor, an extension of HOG targeting the description of light-field imaging disparity variations. Raw light-field data are pre-processed with the MATLAB Light-Field Toolbox: each image is represented as a 4-dimension matrix L(u,v,x,y) where the first two values (u,v) indicate the considered sub-aperture and the last two values (x,y) the position of the pixel in the sub-aperture image. The horizontal $G_{x}\left(x,y\right)$ and vertical $G_{y}\left(x,y\right)$ disparity gradients are defined by Equation (17).

(17) $$\left\{\begin{array}{c} G_{x}\left(x,y\right)=L\left(u_{1},v_{1},x,y\right)-L\left(u_{2},v_{2},x,y\right)\\ G_{y}\left(x,y\right)=L\left(u_{3},v_{3},x,y\right)-L\left(u_{4},v_{4},x,y\right) \end{array}\right.$$

The authors empirically prove that the most suitable sub-aperture images to perform PAD are: $\left(u_{1}=15,v_{1}=8\right)$, $\left(u_{2}=1,v_{2}=8\right)$, $\left(u_{3}=8,v_{3}=15\right)$, $\left(u_{4}=8,v_{4}=1\right)$ – where the sub-aperture image in the top-left corner has indexes (1,1). Then, the disparity gradient magnitude ▽I and orientation θ are evaluated as in Equation (18).

(18) $$\left\{\begin{array}{c} \left|▽I(x,y)\right|=\sqrt{G_{x}{\left(x,y\right)}^{2}+G_{y}{\left(x,y\right)}^{2}}\\ \theta \left(x,y\right)=arctan\left(\frac{G_{x}\left(x,y\right)}{G_{y}\left(x,y\right)}\right) \end{array}\right.$$

The quantization, the normalization, and the concatenation are performed following the standard HOG algorithm. Compliant to the ISO/IEC standard [70] they report BPCER at a fixed 1% APCER. Detection accuracy for the set of presentation attack instruments (including laptop, tablet, mobile and paper) ranges from 0% to 0.45%.

**Figure 17:**
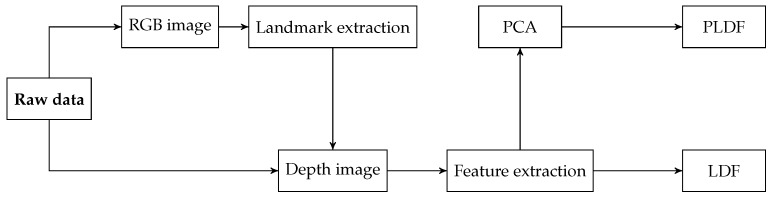
Workflow of the PAD method proposed in [77].

In [77], a PAD method based on RGB-depth image pairs is presented. From each light-field raw file, a 2D-RGB image and a depth map of the captured face are computed. These images are already provided by the IST-EURECOM LFFD, but they can be easily computed from any light-field image. First, face landmark detection is performed on the 2D RGB image, identifying 68 landmarks for each face in the image. Then, depth values associated with each landmark are extracted from the corresponding depth map and used to define a feature vector called landmark depth feature (LDF). To evaluate the advantages of reducing the feature dimension, an alternative feature vector, namely Principal LDF (PLDF), composed by the first 10 principal components, is obtained by applying principal component analysis (PCA) on LDF. Figure 17 illustrates the workflow of the method. The two sets of features, LDF and PLDF, are tested separately. Three experiments show a HTER less than 1% for all the tested protocols, outperforming other PAD algorithms designed for light fields.

Convolutional neural networks (CNN) have been recently tested for PAD on light-field face images by Liu et al. in [24]. The proposed approach is based on two different features: the microlens image and the ray-difference image. The ray-difference image is computed, as described above, using the views at distance 3 from the central view. The 4 error maps obtained are concatenated to be fed into the CNN. The CNN is configured to process an input of size 250×250×3, where 3 corresponds to the RGB color channels. The CNN is implemented on the tensorflow platform. Experiments are carried out on a dataset collected by the authors using a Lytro ILLUM camera and including the following PAIs: printed photo, warped photo, and screen-displayed photo. The results are reported in terms of HTER, and with the microlens feature they reach 0.028%.

Table 8 summarizes the performances of the methods described in this section.

What is lacking for light-field PAD research is a comparative benchmark on a database that includes sophisticated silicone mask [78], which should be considered now state of the art in face presentation attacks.

## 6. Conclusions

As soon as new visual sensors are placed on the market, it is usual that several R&D teams revisit existing methods to process the new image formats in terms of transmission, compression, and displaying. It was the case, some years ago, for Microsoft Kinect imaging sensors [79]. The Joint Photographic Experts Group (JPEG) Standardization committee has defined the JPEG Pleno to provide a standard framework for representing new imaging modalities, such as texture-plus-depth, point cloud, holographic imaging, and light fields.

Apart from the need for purely adapting existing processing methods to the new image format, it is also of interest to investigate to what extent such new visual sensors, which carry richer information and provide additional features, can increase the performance of some existing tasks related to computer vision, e.g., facial image processing. Recently, some companies (e.g., Lytro, Raytrix) have developed imaging sensors integrating light-field technology, the so-called plenoptic camera. This camera can capture in one shot multiple views of the scene, offering new capabilities in terms of depth estimation and image refocusing.

The present article reviews methods that deal with face image processing including landmark detection, recognition, and presentation attack detection in the context of acquisitions performed by Lytro cameras (almost all existing databases have been collected using Lytro products). It has been proven that the richer information provided by such new sensors offers new capabilities in several existing domains. Regarding face recognition, the use of light-field imaging can contribute to increasing performance but with a non-negligible additional computation complexity. As showed in Table 5, Table 6 and Table 7, the performance gain ranges from 0.53% to 5.7%. At the moment, it is hard to predict the future of such new devices in real applications for face recognition.

The results obtained from the application of light fields for face presentation attack detection are encouraging. The provided depth information makes light-field-based PAD solutions robust to 2D presentation attacks instruments. This is of particular importance in unsupervised authentication scenarios, where the attacker can easily present a 2D replica of an authorized user face without being detected. However, as previously mentioned in Section 5, it would be of great interest to investigate more sophisticated PAIs, such as silicone masks. If we consider other fields of research, light fields have proven to be useful for accurate millimetric measurement for automated visual inspection. In [80] the use of refocused depth calibrated images allows performance of measurements on industrial objects and the authors also show that a pixel metric scale can be estimated at different depths, avoiding the use of other measurement devices. It would be interesting to see if these techniques could be successfully applied for PAD in presence of 3D face masks.

Although light fields have not been applied in this field yet, the authors believe that a promising employment of such technology is for facial expression or micro-expression analysis. Section 3 has illustrated the possible benefits of using light-field images for more accurate face landmark detection. The use of light-field videos would also allow the study of the dynamic of the landmarks while the face changes expression.

Light-field devices able to record videos have already been deployed by Raytrix (Read more at https://raytrix.de/). It is safe to assume that in the future more devices will be developed and that they will integrate more features, such as near infrared and thermal imaging sensors. The computational complexity of light-field pictures comes also from the number of microlenses in the microlens array mounted on the plenoptic camera. Some tasks may require the use of only a small number of views. Some preliminary results obtained in [80,81] lead to reduce the number of views. A custom prototype of light-field camera is obtained by putting a small array of microlenses (e.g., of size 2×2) in front of the imaging sensor. In this case, 3D information is sparse but preserved. Concerning Lytro, to date the American company has ceased operations in late March 2018.

Finally, as demonstrated by this survey, light-field face analysis so far has been assessed almost only in comparison to 2D face analysis. A future line of study could therefore investigate the benefits of light fields for face analysis when compared to other imaging technologies, including but not limited to texture-plus-depth, point cloud, thermal, and high dynamic range.

## Figures and Tables

**Table 1:** General-purpose light-field databases that include facial images.

Name	Year	Image Acquisition	Spatial Resolution	Images with Faces	Content Variations
The (New) Stanford Light-Field Archive [25]	2008	Camera array	45×640×480	1 (students behind bushes)	Pose; distance; multiple people; occlusion; outdoor
EPFL Light-Field Image Dataset [26]	2016	Lytro Illum	15×15×625×434	18 (category: people)	Pose; distance; multiple people; outdoor
Stanford Lytro Light-Field Archive [27]	2016	Lytro Illum	15×15×625×434	17 (category: people)	Pose; distance; multiple people; occlusion; outdoor
SMART [28]	2016	Lytro Illum	15×15×625×434	1 (person)	Close-up, one person; reflection; indoor
Technicolor Light-Field dataset [29]	2017	4×4 camera rig	4×4×2048×1088	Several	Pose; distance; indoor

**Table 2:** Light-field face (and facial component) databases.

Name	Year	Image Acquisition	Image Type	Spatial Resolution	Test Subjects	Content Variations
GUCLF [30]	2013	2D camera; Lytro	2D; 2D rendered	5184×3456; 120×120	25	Pose; distance; illumination, multiple people; indoor; outdoor
LFC-MFD [31]	2016	2D camera; Lytro	2D; 2D rendered	5184×3456; 120×120	112	Pose; distance; illumination, multiple people; indoor; outdoor
IST-EURECOM LFFD [32]	2017	Lytro Illum	4D light field; 2D rendered; 2D depth map	15×15×625×434; 2022×1404; 2022×1404	100	Multiple sessions, pose, illumination, expression, occlusion
LFC-VID [31]	2016	Lytro	2D rendered	1080×1080	55 (iris)	Distance range: 9–15 inches; indoor
LLFEDB [33]	2018	Lytro Illum	4D light field; 2D rendered	15×15×625×434; 2022×1404	67 (ear)	Multiple sessions, pose, illumination, occlusion

**Table 3:** Light-field face presentation attack detection databases.

Name	Year	Image Acquisition	Image Type	Spatial Resolution	Test Subjects/Images	Attack Instruments
GUC-LiFFAD Database [34]	2015	Lytro	2D rendered (various depth/focus)	1080×1080	80 subjects/80 *bona fide* images; 2400 2D attack images	Printed paper: laser + inkjet, tablet
LLFFSD [35,36]	2018	Lytro Illum	4D light field; 2D rendered; 2D depth map	15×15×625×434; 2022×1404; 2022×1404	50 subjects/100 *bona fide* images; 600 attack images	Printed paper, wrapped printed paper, laptop, tablet, smartphone 1, smartphone 2
LLFEADB [37]	2018	Lytro Illum	4D light field; 2D rendered	15×15×625×434; 2022×1404	67 subjects/268 *bona fide* images; 1072 attack images	Laptop, tablet, smartphone 1, smartphone 2

**Table 4:** Face landmark correction results.

	Neutral Frontal Face		Action Mouth Open		Pose Up Looking		Pose Half-Profile Left	
	Original	Corrected	Original	Corrected	Original	Corrected	Original	Corrected
**P(%)**	97.81	**98.11**	95.70	**96.37**	91.80	**92.66**	77.68	**79.13**

**Table 5:** Summary of multi-focus-based face recognition methods. Performance is assessed in terms of identification rate (IR) for all methods except for [61], where the equal error rate (EER) is reported. The abbreviations used in this table: WE–wavelet energy image selection; LBP–local binary pattern; LG–log-Gabor filter; LE–log-entropy image selection; SP–super-resolution; LE–log-entropy; LP–Laplacian pyramid image fusion; SRC–sparse reconstruction classifier; SRC–sparse reconstruction classifier; GUCLF–GUC light-field database.

Ref.	Year	Feature Extractor	Classifier	LF DB	2D Baseline	LF Perf.	Gain
[5]	2013	WE; LBP; LG	SRC	GUCLF	75.53% IR	79.10% IR	3.57%
[53]	2013	SR; LBP	SRC	GUCLF	-	53.62% IR	-
[59]	2013	LE; LBP; LG	SRC	GUCLF	-	75.12% IR	-
[60]	2013	LBP	SRC	GUCLF	-	60.56% IR	-
[61]	2015	LP; LBP	SRC	GUCLF	-	4.14% EER	-

**Table 6:** Summary of sub-aperture-based face recognition methods. The abbreviations used in this table: IR—identification rate; ACC—accuracy; LFLBP—light-field local binary pattern; OF—OpenFace; NN—nearest neighbor; $D_{min}$—minimum distance; LFFD—IST-EURECOM light-field face database.

Ref.	Year	Feature Extractor	Classifier	LF DB	2D Baseline	LF Perf.	Gain
[62]	2017	LFLBP	NN	LFFD	89.1% IR	92.1% IR	3%
[63]	2018	OF	$D_{min}$	LFFD	99.27% ACC	99.80% ACC	0.53%

**Table 7:** Summary of deep-learning-based face recognition methods. Abbreviation used in this table: IR–identification rate; VGG-Face–Visual Geometry Group’s CNN descriptor; LSTM–long short-memory recurrent network; SVM–support vector machine; LFFD–IST-EURECOM Light-Field Face Database.

Ref.	Year	Feature Extractor	Classifier	LF DB	2D Baseline	LF Perf.	Gain
[66]	2018	VGG-Face	SVM	LFFD	96.8% IR	98.1% IR	1.3%
[68]	2018	VGG-Face + LSTM	SoftMax	LFFD	92.90% IR	98.60% IR	5.7%

**Table 8:** Summary of presentation attack detection methods. Abbreviations used in this table: EF—edge feature; RD—ray-difference image; FV—focus variation; DR—decision rule; LFHOG—light-field histogram of oriented gradients; LFALBP—light-field angular local binary pattern; HDG—histogram of disparity gradients; LDF—landmark depth feature; PLDF—principal landmark depth feature; CNN—convolutional neural networks; SVM—support vector machine; GUC-LiFFAD—GUC Light-Field Face Artefact Database; IST LLFFSD—IST Lenslet Light-Field Face Spoofing Database.

Ref.	Year	Feature Extractor	Classifier	LF DB	LF Perf.
[74]	2014	EF + RD	SVM	Private	0.89–4.22% HTER
[34]	2015	FV + DR	SVM	GUC-LiFFAD	4.01–5.27% HTER
[75]	2016	LFHOG	SVM	Private	99.75% ACC
[35]	2018	$LFALBP_{HVS}$ + $LFALBP_{YC_{b}C_{r}}$	SVM	IST LLFFSD	0.33–2.85% HTER
[36]	2018	HDG	SVM	IST LLFFSD	0–0.45% BPCER @ 1%APCER
[77]	2018	LDF; PLDF	SVM	IST LLFFSD	0–0.8% HTER
[24]	2019	CNN	SVM	Private	0.028% HTER
